# DNA/RNA hybrid profiling in autistic patients: A focus on mRNA and non-coding RNA variations

**DOI:** 10.1371/journal.pone.0326901

**Published:** 2025-11-03

**Authors:** Leila Kianmehr, Kasra MokhtarZadeh, Zeynep Yilmaz, Hadi Darzi Ramandi, Ecmel Mehmetbeyoglu Duman, Elif Funda Sener, Serpil Taheri, Minoo Rassoulzadegan

**Affiliations:** 1 Betul Ziya Eren Genome and Stem Cell Center, Erciyes University, Kayseri, Turkey; 2 Department of Medical Biology, Medical Faculty, Erciyes University, Kayseri, Turkey; 3 Animal Sciences and Marine Biology Department, Faculty of Life Sciences and Biotechnology, Shahid Beheshti University, Tehran, Iran; 4 Department of Molecular Physiology, Agricultural Biotechnology Research Institute of Iran, Agricultural Research Education and Extension Organization (AREEO), Karaj, Iran; 5 ISERM-CNRS, Université de Nice, Nice, France; University of Cincinnati College of Medicine, UNITED STATES OF AMERICA

## Abstract

Autism spectrum disorder (ASD) is a set of genetically heterogenous neurodevelopmental disorders characterized by core symptoms including impaired social interaction, communication deficits, and restricted or stereotyped behaviors. While a significant number of cases are not explained by Mendelian inheritance, there is growing evidence for implication of non-coding RNAs (ncRNAs) in the development and inheritance of ASD. Transcriptional studies often face challenges due to patient-specific variations in gene expression and technical differences in preserving RNA integrity. We propose that isolating RNA from DNA/RNA hybrids provides a robust method to reliably capture transcriptional information. We performed a whole transcriptome analysis on blood samples from ASD patients and healthy controls to investigate transcripts associated with DNA/RNA hybrids. We identified 278,300 novel transcripts across 68,487 DNA/RNA hybrid loci, with significant enrichment in exonic and intronic regions. The novel long non-coding RNAs (lncRNAs) we found showed higher expression levels compared to known transcripts. Differential expression analysis revealed 301 significantly upregulated and 401 downregulated known transcripts in ASD samples compared to controls (|log_2_-fold change| > 1 and adjusted *p-value* < 0.05). Through qRT-PCR validation, we confirmed the significant upregulation of *RN7SK* and *SMARCC2* associated with DNA/RNA hybrids in ASD patients. Pathway and enrichment analyses highlighted mitochondrial dysfunction and energy metabolism. Our results suggest that ncRNAs can form DNA/RNA hybrids that influence gene expression, providing preliminary insights into the mechanisms of transcriptional dysregulation in ASD.

## Introduction

Autism spectrum disorder (ASD) is a complex group of neurodevelopmental disorders characterized by impaired social interaction and communication, as well as the presence of restricted and repetitive behaviors [1]. The prevalence of ASD is increasing globally, yet its underlying mechanisms remain poorly understood. Both genetic and non-genetic factors as well as their interactions are implicated in the etiology of ASD, leading to altered brain development and neural activity [2,3].

While large-scale exome sequencing studies have identified hundreds of genes associated with ASD [4], no consistent, uniform genetic signature has emerged. Only a small fraction of cases is linked to monogenetic disorders such as Fragile-X syndrome and Rett syndrome [5]. For the majority, the genetic cause remains often uncertain because of complex genetic variations in human. Previous research has consistently highlighted aberrant gene expression patterns in various tissues of autistic individuals, including cerebral cortex and peripheral blood lymphocytes [6–8]. These studies reveal that transcriptional dysregulation in ASD often exhibits significant tissue-specific differences [6–8], such as directional changes in inflammatory genes observed between the brain and blood [6].

While transcriptional variation is a key feature in ASD, a major challenge is the variability observed among the patients. This inconsistency can partly stem from technical differences in how studies are performed. Since RNA can degrade or fragmented during standard sample preparation, a robust methodology is required to preserve transcriptional integrity. Isolating RNA specifically from DNA/RNA hybrids may offer a technique that better preserves this transcriptional information. DNA/RNA hybrids are present in normal cellular physiology, formed during transcription when the nascent RNA remains temporarily bound to its DNA template [9]. Dysregulation in their abundance and localization is associated with various human pathologies [10–13], including neurodevelopmental and neurodegenerative disorders. Specifically, the aberrant formation of these hybrids, particularly within repetitive genomic regions like telomeres and centromeres, has been linked to genomic instability [14,15]. However, their precise role in ASD remains largely unexplored.

Excessive DNA/RNA hybrid formation, particularly within repeated motifs in post-mitotic neurons, can potentially enhance transcription activity, leading to DNA damage and genomic instability. This can pose a physical block to transcription, leading to gene silencing, as seen in disorders associated with trinucleotide repeat expansions like Fragile-X syndrome [16–18]. Moreover, mutations in genes encoding proteins essential for resolving these structures, such as *SETX* (which encodes Senataxin) and *RNaseH2*, are linked to a global increase in hybrids and are implicated in neurodegenerative conditions [19–22]. We have also previously shown a heritable downregulation of miRNAs in autistic patients and their families, suggesting the involvement of non-Mendelian inheritance mechanisms in ASD [23].

Given the challenge of inconsistent results from traditional studies, we hypothesized that variations in DNA/RNA hybrid profiles could provide a better view of the transcriptional dysregulation observed in this disorder. To test this, we utilized a novel, antibody-independent method [24–26] to isolate and profile genomic regions associated with these hybrid structures. In this study, we applied this approach to blood samples from individuals with ASD and healthy controls. Using an integrated bioinformatics and molecular biology approach, we performed whole transcriptome analysis to identify differences in both known and novel transcripts associated with DNA/RNA hybrids. The results reveal altered DNA/RNA hybrid profiles in both messenger RNA (mRNA) and long non-coding RNA (lncRNA) regions in autistic patients, offering foundational perspectives on the potential molecular mechanisms underlying ASD.

## Materials and methods

### Study design

Blood samples were collected from both autistic patients and healthy controls following the acquisition of parental consent. Written informed consent was obtained from all parents before their children participated in the study. The cohort consisted of six patients diagnosed with ASD who presented to the School of Medicine Hospital of Erciyes University, along with six healthy individuals without known medical conditions, all of whom provided informed consent. The diagnosis of autism was confirmed by a multidisciplinary team, including an experienced child psychiatrist, a paediatric neurologist, and a genetic specialist. Diagnostic criteria were based on the Fourth and Fifth Editions of the Diagnostic and Statistical Manual of Mental Disorders according to DSM-IV-TR, and DSM-V criteria [27,28], utilizing the Childhood Autism Rating Scale (CARS) [29]. All participants underwent comprehensive screening for signs of infection, and individuals with acute illnesses were excluded to ensure the integrity of the blood samples and the validity of the research findings. The patient cohort included participants with a range of symptoms and family histories:

- **Patient 1:** A family history of schizophrenia in the paternal grandmother.
- **Patient 2:** A family history of autism and a male sibling with the disorder.
- **Patient 3:** A family history of Asperger syndrome, an uncle with schizophrenia, and a family history of speech delay.
- **Patient 4:** A history of intellectual disability, a sibling with autism, and epilepsy/seizures, with consanguinity present in the family.
- **Patient 5:** A history of intellectual disability and a sister with similar neurodevelopmental features. The patient’s development halted after a febrile seizure at 8 months, and there is third-degree consanguinity in the family.
- **Patient 6:** A small cerebellum, mild intellectual disability, and a paternal cousin with a history of seizures and autism. Consanguinity is also present in the family.

Blood samples were obtained from six patients and six healthy individuals between January 2009 and December 2010. All participants were from the same ethnic background, which was explicitly stated as Turkish families from the Anatolian region. Clinical characteristics of the samples are summarized in S1 Table. This study was approved by Ethics Committee of the Erciyes University School of Medicine (Committee No: 2011/10, approval date: 09-20-2011).

### DNA/RNA hybrid preparation

We used a previously developed method to isolate DNA/RNA hybrids from total nucleic acids without relying on antibodies, which minimizes potential artifacts and enhances reproducibility [29–31]. This approach modifies the classical TRIzol-chloroform extraction protocol to specifically fractionate and purify DNA/RNA hybrids without the need for antigenic recognition or *RNaseH* treatment.

Following the enzymatic removal of proteins, total nucleic acids were isolated and then fractionated using the standard TRIzol^TM^ protocol [30]. The two primary fractions, the aqueous phase (containing free RNA) and the chloroform-water interphase (containing genomic DNA and DNA/RNA hybrids), were separately precipitated with ethanol. The interphase material, containing the target hybrid, was subsequently purified using Zymo-SpinTM columns (Zymo Research Corp, Irvine, CA, USA) or chloroform extraction. To resolve the hybrid structures from residual proteins, the ethanol-precipitated interface material was treated overnight at 56°C with a buffer (20 mM Tris (pH 8), 50 mM EDTA, 0.5% SDS, 20 µM dithiothreitol, and 400 µg/mL Proteinase K). The resulting DNA/RNA hybrid-containing fraction was then subjected to DNase digestion to remove unbound genomic DNA, followed by additional column purification. This multi-step process isolated the RNA component specifically bound within the hybrid structures, separating it from the free RNA fraction. Finally, the presence of isolated DNA/RNA hybrid fraction was confirmed by testing the sensitivity of extracts to *RNaseH* (an enzyme specific for degrading the RNA strands of a DNA/RNA hybrid) and DNase (to complete removal of remaining genomic DNA fragments).

### RNA sequencing

A quantity of 10–100 ng of RNA was recovered from blood samples after DNase digestion. RNA libraries were prepared from biological replicates of the DNA/RNA hybrid-bound RNA from the autistic patients and healthy controls. High-throughput sequencing was performed using the Illumina HiSeq 2500 or MiSeq platform at Eurofins Medigenomix GmbH (Ebersberg, Germany).

### Bioinformatics analysis

Quality control of the raw sequencing data was performed using *FastQC* (version 0.11.9) and *Trimmomatic* (version 0.39) to remove low-quality reads and adapter sequences [31]. This process involved removing 12 nucleotides from the 5′ end of each read and trimming bases with a Phred score below 20 from both 5′ and 3′ ends. Reads shorter than 35 base pairs after trimming were discarded. Trimmed reads were aligned to the human reference genome (GRCh38) using *HISAT2* [32] with the command: ‘hisat2 --dta --rna-strandness’. *Samtools* was used for sorting and indexing alignment files.

### Transcriptome assembly

Potential ribosomal RNAs and reads not aligned to the reference genome were filtered out using *RseQC* tool (version 4.0.0) [33]. Genome-guided transcriptome assembly was performed using *StringTie* (version 2.1.1) [34], using the command `stringtie –rf` with the annotation file in GFF3 format (gencode.v39.chr_patch_hapl_scaff.annotation.gff3) obtained from the GENCODE database (https://www.gencodegenes.org/human). For each sample, an Individual transcriptome was generated and merged using the *StringTie* merge command to create a reference transcriptome assembly for quantification. Strand-specific settings were applied throughout both the alignment and assembly processes. Gene expression quantification was conducted using *HTSeq-count* (version 0.11.2) [35]. For visualization, the *Integrative Genome Viewer* (*IGV*, version 2.12.0) was used [36]. To facilitate comparative visualization, reads were normalized across samples to 10^7^ reads per sample.

### Differential gene expression (DGE) analysis

DGE analysis was performed using *DESeq2* (version 1.36.0) [37]. To account for multiple testing, *p*-values were adjusted for multiple comparisons using the Benjamini-Hochberg method. Transcripts with an absolute log_2_-fold change greater than 1 and an adjusted *p*-value of less than 0.05 were considered significantly differentially expressed. Gene ontology (GO) and KEGG pathway over-representation analyses were performed using the *Cluster Profiler* package (version 4.4.4) [38] and the org.Hs.e.g.,db annotation package (version 3.15.0).

### Detection of novel lncRNAs

Novel transcripts were identified using *GffCompare* (version 0.12.6) [39], focusing on unannotated transcripts originating from intergenic and antisense regions with a minimum length of 200 nucleotides. These transcripts were subsequently screened for coding potential. Nucleotide sequences were extracted using *SeqKit* [40] and *GffRead*. The coding potential of each transcript was evaluated using the *Coding Potential Assessment Tool* (*CPAT*), which employs a logistic regression model to predict coding potential from nucleotide sequences [41]. The highest coding potential was determined across all identified open reading frames (ORFs) for each transcript. Transcripts with a coding probability score below a threshold of 0.364 were classified as novel lncRNAs.

### Peak finding

Peak identification was performed using *HOMER* (version 4.11) [42] to detect significantly enriched peaks, which represents genomic region-specific associations of DNA/RNA hybrids. The analysis was conducted using the commands: ‘findpeaks *–o auto’* and ‘*annotatePeaks.pl’*. A False Discovery Rate (FDR) threshold of 0.001 was applied. Peaks were then annotated to genomic features, including promoter-TSS (Transcription Start Site), 5’ UTR (Untranslated region), exon, intron, 3’ UTR, TTS (Transcription termination site), intergenic, and non-coding regions, based on the GRCh38 reference genome.

### cDNA synthesis and qRT-PCR

Total RNA samples were reverse transcribed into complementary DNA (cDNA) using the Evoscript Universal cDNA Master Kit (Roche, Mannheim, Germany, Cat No: 07912439001), in a final reaction volumes of 20 µL following the manufacturer’s protocol. cDNA samples were stored at −80 °C until further analysis. qRT-PCR validation was performed on six significantly differentially expressed transcripts using the LightCycler 480 II high-throughput Real-Time PCR system (Roche, Mannheim, Germany). Prior to amplification, cDNA samples were diluted in 1:5 in nuclease-free water. SYBR Green Master Mix (Roche, Mannheim, Germany, Cat No: 04707516001) was used to quantify transcript levels of lncRNAs (*SLC12A5-AS1*, *RN7SK*), and protein-coding genes (*SLC16A3, NLGN3, SMARCC2* and *ADAMTSL4),* using *GAPDH* as the reference gene. The reaction mix was prepared according to the manufacturer’s instructions. Relative gene expression changes were calculated using the 2^−ΔΔCt^ across all experimental groups [43].

### Statistical analysis and visualization

Data visualization and statistical analyses were performed using *R* software (version 4.3.3). Heatmaps were generated with the *pheatmap* package (https://cran.r-project.org/package=pheatmap) [44], while additional visualizations were created using *ggplot2* [45] and *ggrepel* packages. Data were normalized to a standardized scale ranging from 0 to 1 before generating heatmaps. Various statistical tests─including the *t-test*, *chi-square* test, and *Fisher’s* Exact test─were utilized for variance analysis, with the specific test selected based on the characteristics of the data and the research objectives. All statistical analyses were executed within the *RStudio* environment, with statistical significance established at an adjusted *p-value* < 0.05.

## Results

We investigated the transcriptome profiles of DNA/RNA hybrids in blood samples from individuals with ASD and healthy controls to reveal the potential transcriptional alterations in the disorder. Applying our previously developed methodology for analyzing DNA/RNA hybrid fractions, we established a robust framework for transcriptome analysis. Specifically, we sought to identify differences in hybrid formation that may contribute to ASD pathology.

**Fig 1:**
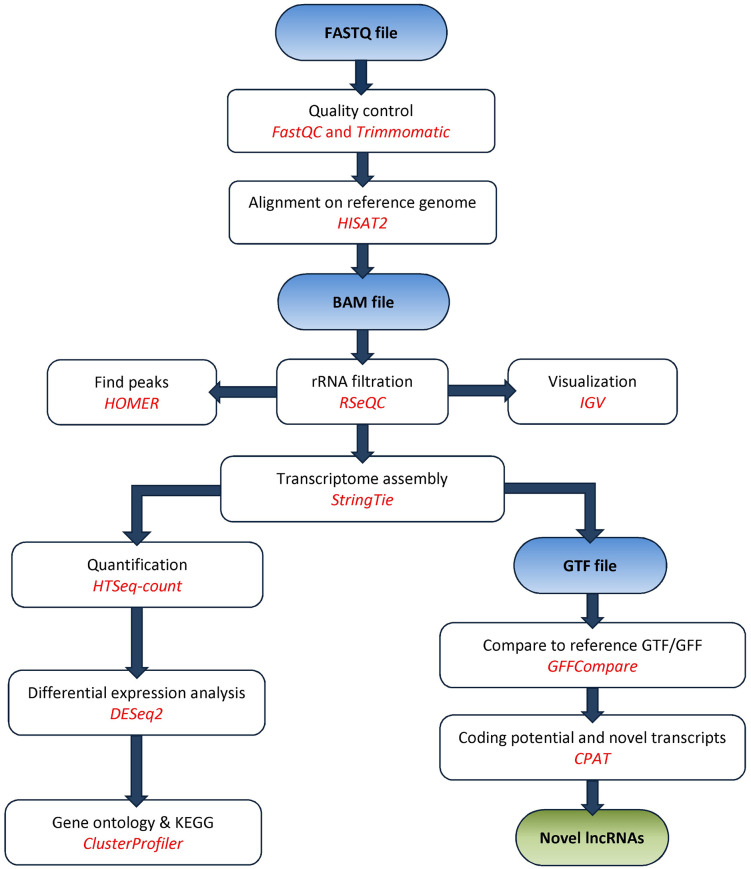
Schematic workflow for the identification DNA/RNA hybrid-associated transcripts and novel lncRNAs in ASD. The pipeline illustrates the bioinformatic analysis steps: 1. Quality control and alignment: raw FASTQ files undergo quality control (FastQC) and trimming (Trimmomatic). Processed reads are aligned to the human reference genome (GRCh38) using HISAT2 to generate BAM files. 2. Transcriptome assembly and quantification: Ribosomal RNA contamination is filtered (RSeQC). Genome-guided transcriptome assembly is performed (StringTie), and gene expression levels are quantified (HTSeq-count). 3. DGE analysis is conducted (DESeq2). Functional enrichment analysis, including Gene Ontology and KEGG, is performed (ClusterProfiler). 4. Novel transcript identification: Assembled transcripts are compared to reference annotations (GFFCompare) to identify novel transcripts, which are then screened for coding potential (CPAT) to identify novel lncRNAs. 5. Peak finding and visualization: Genomic region-specific associations of DNA/RNA hybrids are identified (peak finding) using HOMER. RNA signals are visualized using IGV.

A total of 3.5 × 10⁸ total paired-end reads were generated. Due to quality control and read coverage requirements, the final analysis was conducted using five samples (three ASD patients and two healthy controls). Genomic mapping of RNA-seq reads was performed using the human GRCh38 genome assembly, as summarized in the schematic pipeline (Fig 1). Following quality control and mapping, uniquely mapped paired-ends reads were used for genome-guided transcriptomes assembly.

### Identification of novel transcripts associated with DNA/RNA hybrids

Genome-guided transcriptome assembly identified 278,300 transcripts distributed across 68,487 DNA/RNA hybrid loci. The high support scores for the generated transcripts reflect the high sensitivity and accuracy of the assembly process (S2 Table). This assembly revealed 9,501 novel exons and 2,930 novel introns spanning 5,196 genomic loci. The biotype analysis of known transcripts showed 49% were protein-coding, 36% were lncRNAs, and 15% were pseudogenes-derived transcripts (S1 Fig).

### Enrichment of DNA/RNA hybrids in genomic regions of ASD blood cells

**Fig 2:**
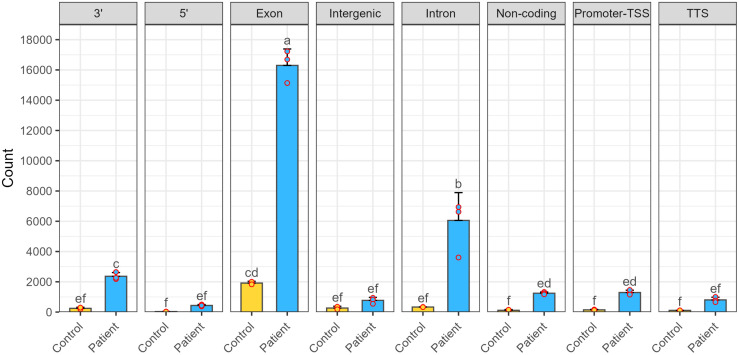
The average percentage of significant DNA/RNA hybrid-associated peaks across different genomic regions in ASD patients (n = 3) and healthy controls (n = 2). Genomic regions annotated include the promoter-TSS (Transcription Start Site), 5’ UTR (Untranslated Region), exon, intron, 3’ UTR (Untranslated Region), TTS (Transcription Termination Site), intergenic, and non-coding regions. Peaks are notably enriched in the exonic and intronic regions of ASD patients compared to controls. Error bars represent the mean ± Standard Deviation (SD). Statistical comparisons were performed for each genomic region using One-way ANOVA followed by Duncan’s multiple comparison test (*p*-value < 0.05).

Genome-wide distribution analysis using HOMER evaluated the distribution of DNA/RNA hybrids across genomic features, including the promoter-TSS, 5’ UTR, exons, introns, 3’ UTR, TTS, intergenic, and non-coding regions. DNA/RNA hybrids were enriched across all genomic regions; however, with the most significant enrichment observed in exonic and intronic regions (Fig 2). When comparing patient groups, the ASD blood cells exhibited a pronounced and significant accumulation of DNA/RNA hybrids within the exonic and intronic regions compared to control blood cells.

### Differential expression and identification of novel lncRNAs associated with DNA/RNA hybrids

**Fig 3:**
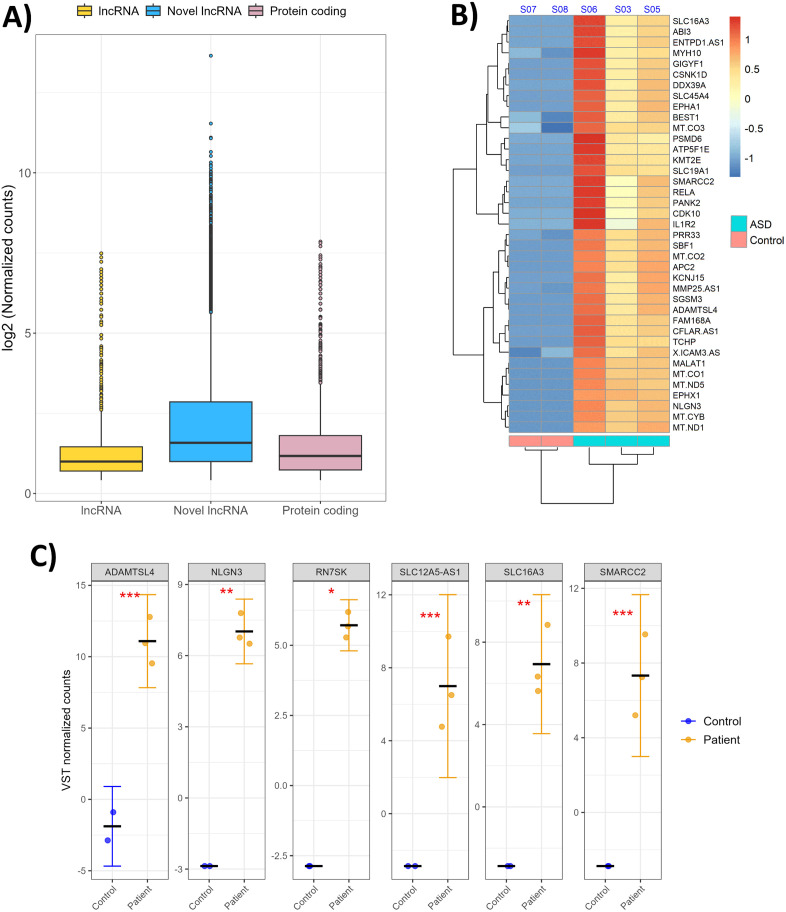
Biotype and differential expression analysis of transcripts identified in ASD and control samples. (A) Distribution of expression levels of lncRNAs, novel lncRNAs and protein-coding transcripts across all samples. (B) Heatmap of the most significantly differentially expressed transcripts comparing ASD patients to healthy controls. Color intensity represents expression level (Z-score of log-normalized counts), with yellow/orange indicating high expression (log2-fold change >1, adjusted *p*-value <0.05) and blue indicating low expression (log2-fold change <−1, adjusted *p*-value <0.05). (C) Expression levels of selected candidate transcripts. Jitter plots represent the variance stabilized transformed (VST) normalized counts for key selected DE transcripts: protein-coding genes (*ADAMTSL4*, *NLGN3, SLC16A3*, and *SMARCC2*) and lncRNAs (*RN7SK, SLC12A5-AS1*). Each dot represents an individual sample and mean ± standard error (SE) is shown. Statistical significance is indicated as follows: *p *< 0.001(***), *p *< 0.01(**), *p *< 0.05(*). All selected transcripts exhibited significantly higher expression in ASD patients than in controls.

Transcriptome profiling of DNA/RNA hybrids-derived transcripts identified 13,305 known transcripts across all samples after filtering out lowly expressed transcripts. Novel lncRNA transcripts exhibited higher expression level compared to both annotated protein-coding genes and known lncRNAs (Fig 3a).

Differential expression analysis (using |log_2_-fold change| > 1 and adjusted *p-value* < 0.05) identified 702 differentially expressed transcripts: 301 transcripts were significantly upregulated and 401 transcripts were significantly downregulated in ASD patients compared to healthy controls (S3 Table). The most significantly upregulated transcripts (based on log_2_-fold change and baseline expression) are illustrated (Fig 3b, S2 and S3 Figs). Among these, several transcripts, including protein-coding transcripts (*ADAMTSL4, NLGN3, SLC16A3,* and *SMARCC2*), and lncRNAs (*RN7SK, SLC12A5-AS1*) exhibited higher expression levels in ASD samples compared to healthy controls (Fig 3c). *ADAMTSL4*, *SMARCC2*, and *SLC12A5-AS1* demonstrated high significance (*p-*value < 0.001). *SLC1A3* and *NLGN3* were significantly different (*p*-value < 0.01), and *RN7SK* showed moderate significance (*p*-value < 0.05).

**Fig 4:**
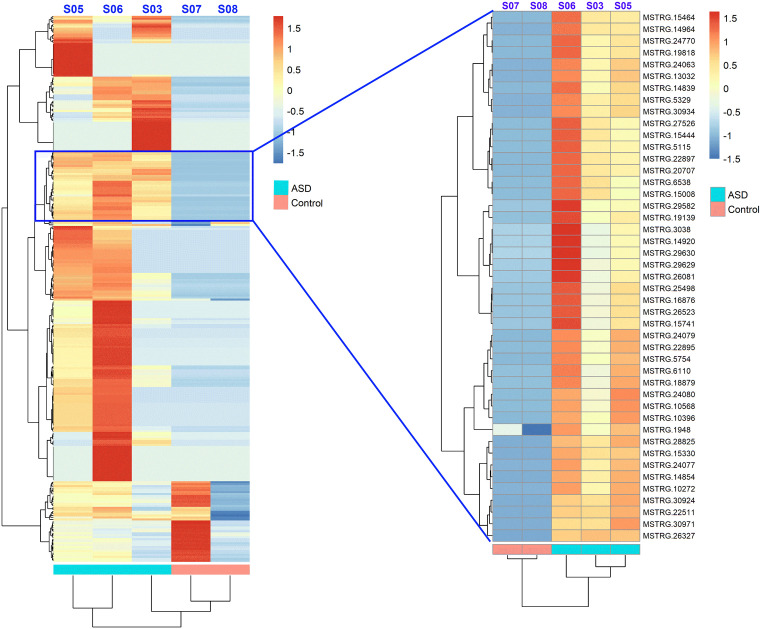
Heatmap visualizing the expression of all identified novel lncRNAs across ASD patients and healthy controls (left). Hierarchical clustering reveals a distinct module containing 45 novel lncRNAs (blue box) which represents a group of transcripts consistently enriched in the DNA/RNA hybrid fractions of the ASD patients samples (right).

To identify novel lncRNAs associated with DNA/RNA hybrids, we analyzed 1,755 previously unannotated transcripts. The CPAT classified these transcripts using a coding probability threshold of 0.364, resulting in the identification of 1,048 novel lncRNAs and 707 protein-coding transcripts (S4 and S5 Figs, S4 Table). All 1048 novel lncRNAs were confirmed as unannotated as they were absent from the GENCODE v39 reference transcriptome. However, none of these novel lncRNAs met the defined threshold for differential expression in this cohort. Analysis of their exon structure revealed that the majority (58.1%) consisted of a single exon, while 36.1% contained two or more exons (S6 Fig). Principal component analysis (PCA) demonstrated separation of variance between the ASD patients and healthy controls (S7 Fig). Furthermore, hierarchical clustering identified a module containing 45 lncRNAs that were consistently enriched across all ASD patient samples (Fig 4).

### Integrative Genomics Viewer (IGV) visualization

**Fig 5:**
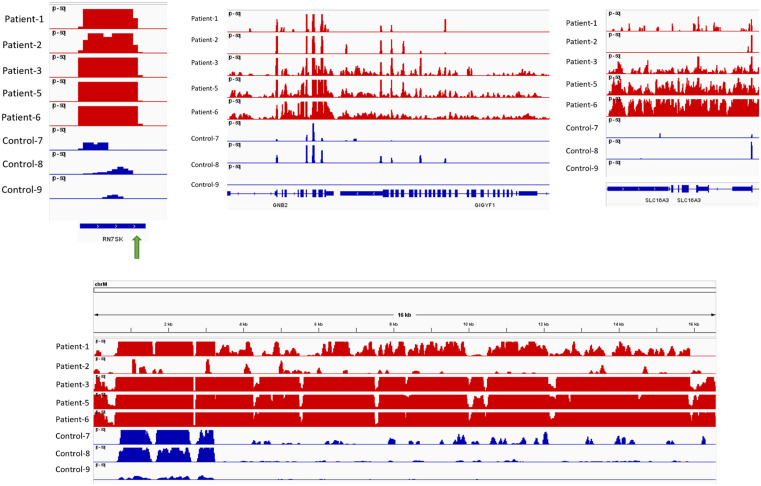
IGV Visualization confirms increased expression signal for selected DNA/RNA hybrid-associated transcripts in ASD, including *RN7SK* (chr6:52,995,773-52,996,031), *GIGYF1*, *SLC16A3,* and mitochondrial genes (*MT-ND5* and *MT-CYB*). ASD patient samples are represented by red tracks and healthy controls by blue tracks. The green arrow indicates the specific genomic loci selected for qRT-PCR validation.

To further explore the functional relevance of differentially expressed transcripts, we cross-referenced our results with the SFARI database (https://gene.sfari.org/), a comprehensive resource cataloging autism-related genetic factors, identifying several transcripts previously reported as associated with autism, including *GIGYF1*, *SMARCC2*, *EPHA1*, *IL1R2*, *KCNJ15*, *KMT2E*, *MYH10*, *NLGN3*, *SBF1*, and *SGSM3* (S5 Table).We further examined the mRNA expression levels of these selected transcripts across various tissues using the GTEx portal (https://gtexportal.org/home/) (S8 Fig). For the IGV visualization, reads from five ASD patients and three healthy controls were utilized. This analysis confirmed strong expression signals in ASD samples compared to healthy controls for key genes such as *SLC16A3* and *GIGYF1* (Fig 5). Furthermore, strong signals observed for other transcripts associated with DNA/RNA hybrids, including *RN7SK* and mitochondrial genes (*MT-ND5* and *MT-CYB*), all of which exhibited significant signal differences in ASD samples compared to healthy controls (Fig 5).

### Functional enrichment and KEGG pathway analysis

To explore the functional roles of differentially expressed transcripts associated with DNA/RNA hybrids in ASD patients (using |log_2_-fold change| ≥ 1 and *p*-value < 0.05), we conducted GO and KEGG pathway analyses.

**Fig 6:**
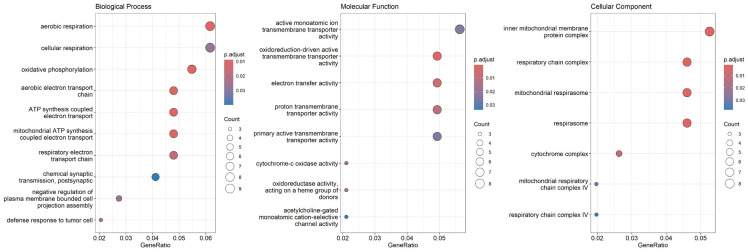
Functional enrichment analysis of differential expressed transcripts using GO. The plots display the most enriched GO terms across BP, MF, and CC. Enrichment is primarily associated with mitochondrial function, the ETC and energy metabolism. The Y-axis represents the enriched GO terms, and the X-axis represents the gene ratio.

**Fig 7:**
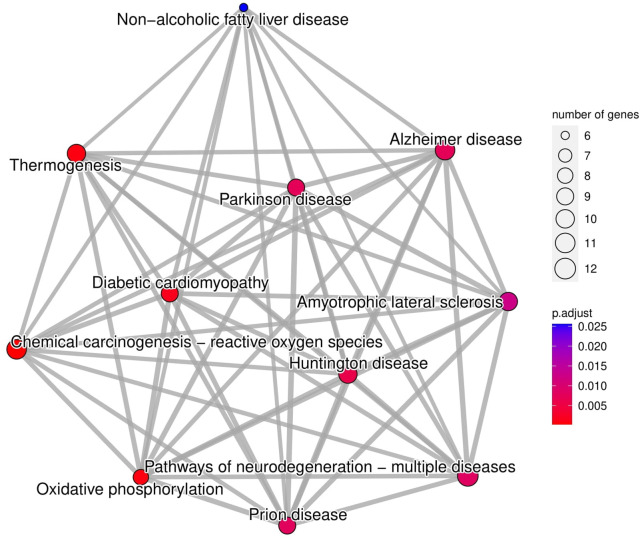
KEGG pathway analysis highlights neurodegenerative disorders pathways and OXPHOS. The size of each circle reveals the number of genes involved in the pathway. The color scale represents the adjusted *p-value*.

The most significantly enriched GO terms were categorized for Biological Process (BP), Molecular Function (MF), and Cellular Component (CC) (Fig 6). The results demonstrated that the transcripts in ASD patients were primarily associated with mitochondrial function, the electron transport chain (ETC), OXPHOS, and energy metabolism (Fig 6). Other relevant GO terms within BP category included neuronal structure and development, such as regulation of plasma cell projection assembly and neuronal active transporters (Fig 6). Additionally, KEGG pathway over-representation analysis revealed a significant enrichment in pathways related to oxidative phosphorylation and multiple neurodegenerative disorders, including Alzheimer’s, Parkinson’s, Huntington’s disease, Amyotrophic Lateral Sclerosis (ALS), and prion disease (Fig 7).

Upon further investigation, key mitochondrial genes were highlighted among these enriched GO terms (S9 Fig), including *ND1* and *ND5* (from ETC complex I), *CYTB* (from ETC complex III), *COX1*, *COX2*, and *COX3* (from ETC complex IV), and *ATP5F1E* (from ETC complex V). Consistent with these results, IGV visualization also showed a high expression signal of mitochondrial genes, including *ND5* and *CYB* in ASD patients compared to controls (Fig 5).

### Validation of RNA-seq results by qRT-PCR analysis

To validate RNA-seq results, we isolated DNA/RNA hybrids-bound RNA and total RNA were from the blood samples of ASD patients and healthy controls. Six transcripts, identified as significantly differentially expressed in the RNA-seq analysis, were selected for validation: **A*DAMTSL4, NLGN3, RN7SK, SLC12A5-AS1, SLC16A3,* and *SMARCC2*. Relative expression levels were quantified using qRT-PCR with *GAPDH* as a reference gene (primers listed in S6 Table).

**Fig 8:**
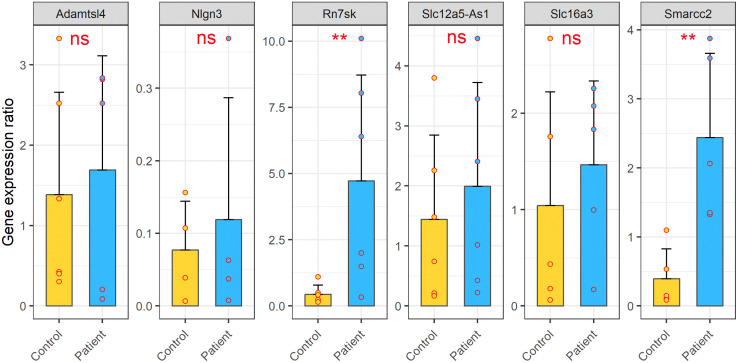
Relative expression levels of six candidate transcripts within DNA/RNA hybrid-bound RNAs fraction from ASD patients compared to healthy controls. Transcripts validated include the lncRNAs *RN7SK and SLC12A5-AS1*, and the protein-coding transcripts *ADAMTSL4, NLGN3, SLC16A3,* and *SMARCC2*. Data are presented as means ± SEM. Statistical significance was determined using a two-tailed, unpaired Student’s t-test (* *p* ≤ 0.05 and ** *p* ≤ 0.01).

In the DNA/RNA hybrids-bound RNA, qRT-PCR results were consistent with the RNA-seq, confirming the increased expression for all six transcripts (Fig 8). Specifically, *RN7SK* and *SMARCC2* showed statistically significant upregulation in ASD patients compared to controls (*p*-value < 0.01). The remaining four transcripts–*ADAMTSL4, NLGN3, SLC12A5-AS1, and SLC16A3–*displayed a similar trend of increased expression but did not reach statistical significance.

**Fig 9:**
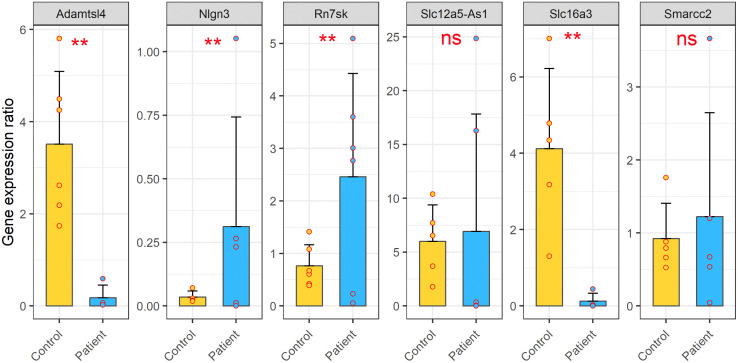
Relative expression levels of six candidate transcripts within total RNAs from ASD patients compared to healthy controls. Transcripts validated include the lncRNAs *RN7SK and SLC12A5-AS1*, and the protein-coding transcripts *ADAMTSL4, NLGN3, SLC16A3,* and *SMARCC2*. Data are presented as means ± SEM. Statistical significance was assessed by two-tailed, unpaired Student’s t-test (* *p* ≤ 0.05 and ** *p* ≤ 0.01).

In contrast, analysis of total RNA fraction showed significant differential expression (means± SEM) for four transcripts: *ADAMTSL4*, *NLGN3, RN7SK,* and *SLC16A3* (*p*-value < 0.01). *NLGN3* and *RN7SK* maintained elevated expression in ASD patients. However, *ADAMTSL4* and *SLC16A3* were downregulated relative to healthy controls (Fig 9). *SMARCC2 and* SLC12A5-AS1 did not show significant differential expression in the total RNA fraction.

## Discussion

The aberrant formation of DNA/RNA hybrids can lead to genomic instability, potentially contributing to neurodevelopmental disorders. However, the mechanisms underlying genome dysregulation in autistic patients remains unclear. In this study, we compared the transcriptomic profiles of DNA/RNA hybrids in ASD patients and healthy controls, hypothesizing that variations in hybrid regions could provide evidence of the molecular mechanisms of disorder.

To investigate whether DNA/RNA hybrid regions are altered in ASD, we employed a robust, previously established methodology [24,25] that enables the recovery of RNA attached to genomic DNA within these hybrid structures. Transcriptome analysis of the resulting DNA/RNA hybrids from blood samples led to identification of over 1,000 previously unannotated/novel lncRNAs. Our results, highlight these novel transcripts associated with DNA/RNA hybrids, which originate from antisense and intergenic regions. Our comprehensive transcriptome assembly included 278,300 transcripts, of which 13,305 were known transcripts expressed across all samples. DGE analysis subsequently revealed 401 significantly downregulated and 301 upregulated known transcripts in ASD patients compared to controls. The pronounced accumulation of DNA/RNA hybrids observed in exonic and intronic regions of ASD blood cells suggests a direct link to altered transcriptional activity in both coding (exonic) and non-coding (intronic) sequences.

The role of lncRNAs has been contentious due to their initially unclear functions. However, accumulating evidence now showing that lncRNAs play critical roles in chromatin remodeling and enhancer regulation during development [46]. This underscores their importance in diverse biological processes. Indeed, dysregulation of ncRNAs has been implicated in the phenotypic changes of various complex disorders, including autism [47], suggesting a significant role for ncRNAs in transcriptional regulation within ASD. Consistent with this, our previous study reported decreased levels of six miRNAs in the immediate family members of autistic patients [23], and other studies have explored ncRNA expression in postmortem brain tissues and mouse models of ASD [48–50], further supporting their involvement in pathogenesis of ASD.

Our results align with the growing recognition that lncRNAs exhibit tissue-specific expression [51] and that structural alterations in DNA/RNA hybrids are important contributors to human diseases [13]. Consistent with this, our prior work has identified variations in DNA/RNA hybrids at telomeric regions and other ncRNAs across multiple pathologies [25,26]. This phenomenon is evident in two ASD-related conditions like Prader-Willi syndrome (PWS) and Angelman syndrome (AS), where complex epigenetic mechanisms underlie disease pathology [52]. This study extends these observations by focusing on dynamic RNA-DNA interactions, which could advance our understanding of cellular and developmental biology, as well as gene-environment interactions.

Recent research highlights the interplay between heredity and *de novo* mutations in autism. While over 100 susceptibility genes have been identified, they account for only a fraction of cases, indicating that they may not fully elucidate the disease aetiology [53]. Increasingly, non-Mendelian inheritance, characterized by predominant variations in ncRNAs, is being recognized as a key contributor. This mechanistic evidence highlights a potential avenue for autism intervention strategies aimed at mitigating risks associated with non-Mendelian inheritance.

Evaluating transcription levels within hybrid regions offers a novel approach to identifying biomarkers and biological pathways that could potentially restore normal gene expression. In this regard, we identified several potential biomarkers, including small nuclear ncRNA *RN7SK* and a subunit of the BAF chromatin remodeling complex *SMARCC2.* While information on *RN7SK* is limited, it has been linked to neuronal development and neurodevelopmental disorders [54,55,56]. Furthermore, *SMARCC2,* is considered a high-confidence ASD candidate [57,56]. A significant enrichment of rare missense variants in *SMARCC2* among individuals with ASD, suggesting a potential genetic susceptibility [58].

Functional enrichment analysis indicated that the majority of enriched GO terms were associated with mitochondrial function and energy metabolism. Mitochondrial dysfunction is associated with various clinical and biochemical abnormalities in individuals with ASD, contributing to the diverse symptoms and comorbidities observed [59]. For example, deficiency of Cytochrome-c oxidase, a key enzyme in the ETC directly impairs energy production [60]. Additionally, dysregulation of plasma cell projection assembly, suggested by our GO analysis indicates defects in cell projections, including axon and dendrite formation, essential for neuronal polarity and circuit formation [61]. These processes may impact synapse formation and neuronal connectivity in ASDs. Other relevant GO terms linked to neuronal active transporters suggest that their impairment disrupts neurotransmitter uptake and ion homeostasis, potentially leading to synaptic dysfunction in neurodevelopmental pathologies [62].

The KEGG pathway over-representation analysis revealed enriched pathways in neurodevelopmental disorders, notably shared common mechanisms with autism. Mitochondrial dysfunction represents a central feature in the development and progression of several neurodegenerative conditions, including Alzheimer’s [63,64], Parkinson’s [65,66], Huntington’s disease, ALS [67], and prion disease [68]. This dysfunction broadly encompasses impaired energy production through perturbed OXPHOS and increased oxidative stress, which contributes to neuronal and synaptic damage, cognitive decline, and overall neuronal degeneration [69]. This shared pathology makes mitochondrial dysfunction and its key component, OXPHOS disruption, a promising target for novel diagnostic and therapeutic strategies across these disorders.

While these results, supported by preclinical proof-of-concept studies, hold promise, additional family-based research is warranted to clarify the role of non-Mendelian inheritance in autism. Phenotypic and disease variations across generations may arise from multigenic influences or non-Mendelian heritable mechanisms, necessitating further investigation under controlled experimental conditions. A key innovation of this work is its focus on “hybrid marks” and the exploration of pathways connecting DNA to RNA. Although the precise pathogenic role of DNA/RNA hybrids remains to be fully elucidated, deciphering their mechanisms may offer novel therapeutic opportunities. Comprehensive profiling of these hybrid regions is essential for future research, particularly in understanding their potential contribution to the transgenerational transmission of neurodevelopmental disorders. This mechanistic view underscores the need for caution in therapeutic applications, particularly given the potential for patient-specific variations in gene expression. To our knowledge, this study provides novel insights into variations of DNA/RNA hybrid-associated ncRNAs as heritable signals in autism, potentially serving as markers for ASD susceptibility.

### Limitations of the study

The primary limitation of this study is the small sample size, which restricted the statistical power needed to ascertain subtle biological differences and limits the conclusive inference of the results. Future validation must be conducted in significantly larger cohort studies to enhance the robustness of results. A second limitation lies in the choice of biological matrix. While the analysis of DNA/RNA hybrid fractions in peripheral blood samples provided a foundational transcriptomic signature, this approach does not fully capture the complexities of non-Mendelian transmission, which are mediated in germline tissues. To more conclusively delineate the precise role of DNA/RNA hybrids in non-Mendelian inheritance, subsequent research must focus on examining these alterations in sperm, egg, or embryo samples. While such experiments present considerable ethical challenges in human subjects, further research incorporating preclinical models, dedicated clinical studies, and family-based experimental designs will be crucial for a comprehensive evaluation of non-Mendelian inheritance mechanisms in autism.

## Conclusions

This work provides novel evidence suggesting a role for DNA/RNA hybrid variations in the molecular mechanisms underlying ASD. Whole transcriptome analysis demonstrated significant alterations of transcripts associated with DNA/RNA hybrids between ASD patients and controls, advancing mechanistic perspective of ASD. This highlights how alterations in hybrid regions, particularly those involving ncRNAs, may modulate gene expression (either enhancing or inhibiting transcription) and may contribute to the pathology of the disorder. The observation of key pathways like mitochondrial dysfunction and signals suggestive of non-Mendelian heritability underscore that dysregulation of DNA/RNA hybrid formation is likely a critical and complex molecular feature contributing to ASD susceptibility.

## Supporting information

S1 Fig RNA biotypes of known transcripts expressed in all blood samples. The left pie chart shows RNA biotypes of all expressed transcripts, while the right pie chart displays RNA biotypes of differentially expressed transcripts.
(TIF)

S2 Fig Number of the most significantly differentially expressed transcripts between autistic patients and healthy controls. The graph is based on normalized transcript counts. Blue and red bars represent autistic patients and healthy controls, respectively.
(TIF)

S3 Fig Volcano plot of differentially expressed transcripts. The plot displays the relationship between log₂ fold change and statistical significance (adjusted p-value). The x-axis represents the log₂ fold change in transcript expression, while the y-axis shows the –log₁₀ of the adjusted p-value. Transcripts with significant differential expression (Padj < 0.05) are highlighted in red for those upregulated in autistic patients (log₂ fold change > 2) and in blue for those downregulated (log₂ fold change < –2).
(TIF)

S4 Fig Distribution of coding probability. Graph shows number of transcripts per coding probability. The dashed red line shows the threshold (0.364). Coding probability below this amount considered non-coding transcripts.
(TIF)

S5 Fig Box plot showing the expression level of known transcripts through all samples. Expression values are log_2_- normalized counts. The top of each bars shows the number of each transcript per genomic regions.
(TIF)

S6 Fig Exonic structure of novel lncRNAs. 94.2% of the lncRNA transcripts had either 1 or 2 exons.
(TIF)

S7 Fig Principal Component Analysis (PCA) plot of samples showing the separation between ASD patients and control based on gene expression profiles. The first principal component (PC1) explains 68.8% of the variance, while the second component (PC2) explains 20.6%. Blue points represent ASD samples (S_03, S_05, S_06), and red points represent control samples (S_07, S_08), indicating distinct clustering between the two conditions.
(TIF)

S8 Fig Bulk tissue gene expression profiles of *SMARCC2* and *GIGYF1* across various tissue types, based on data from the GTEx project. Notably, both genes show significant expression in whole blood.
(TIF)

S9 Fig Genes associated with enriched GO terms in biological processes. Various genes of different mitochondrial electron transport chain complexes are present. The size of each circle representing GO terms indicates the number of genes associated with that term. Fold change (log_2_) is based on the normalized counts of each gene.
(TIF)

S1 Table Clinical characteristics of patients and healthy controls, including age and gender distribution. (PDF)

S2 Table Sensitivity and precision percent across multiple levels of transcripts. (PDF)

S3 Table Differential expression analysis2 revealed 702 differentially expressed transcripts, with 301 transcripts significantly upregulated, exhibiting a higher density of DNA/RNA hybrids in ASD patients (log2-fold change > 1, adjusted P < 0.05), while 401 transcripts were significantly downregulated (log2-fold change < −1, adjusted p < 0.05). (XLSX)

S4 Table Novel lncRNAs based on coding probability < 0.364 from CPAT tools. (Column 1 and 2: seq_ID): provides the identifier of the sequence being analyzed, (Column 3: mRNA): Sequence length of the mRNA or transcript, (Column 3: ORF): Indicates a specific Open Reading Frame (ORF) within the sequence, (Column 4: strand): shows the strand (+ forward, – reverse) of the transcript where the ORF is located, (Column 5: frame): indicates the reading frame (1, 2, or 3) of the ORF, (Column 6: ORF_start): specifies the starting position of the ORF, (Column 7: ORF_end): specifies the ending position of the ORF, (Column 8: ORF), (Column 9,10: ORFFickettn and Hexamer): contain scores related to nucleotide composition bias (Fickett) and hexamer usage bias, (Column 11: Coding_prob): presents the final probability score (between 0 and 1) of the ORF being protein-coding.
(XLSX)

S5 Table List of ASD-related genes among the DEGs, previously reported in the SFARI database as being associated with autism. The cause of the genetic association of each gene with ASD and the number of reports for each gene in relation to ASD is also present.
(PDF)

S6 Table List of primers sequencing nucleotides forward and reverse for Real-Time PCR. (PDF)
