# Supplementary figures and images for: DNA/RNA hybrid profiling in autistic patients: A focus on mRNA and non-coding RNA variations
Source: PLoS One. 2025 Nov 3;20(11):e0326901. doi: 10.1371/journal.pone.0326901 (PMC12582435; doi:10.1371/journal.pone.0326901)

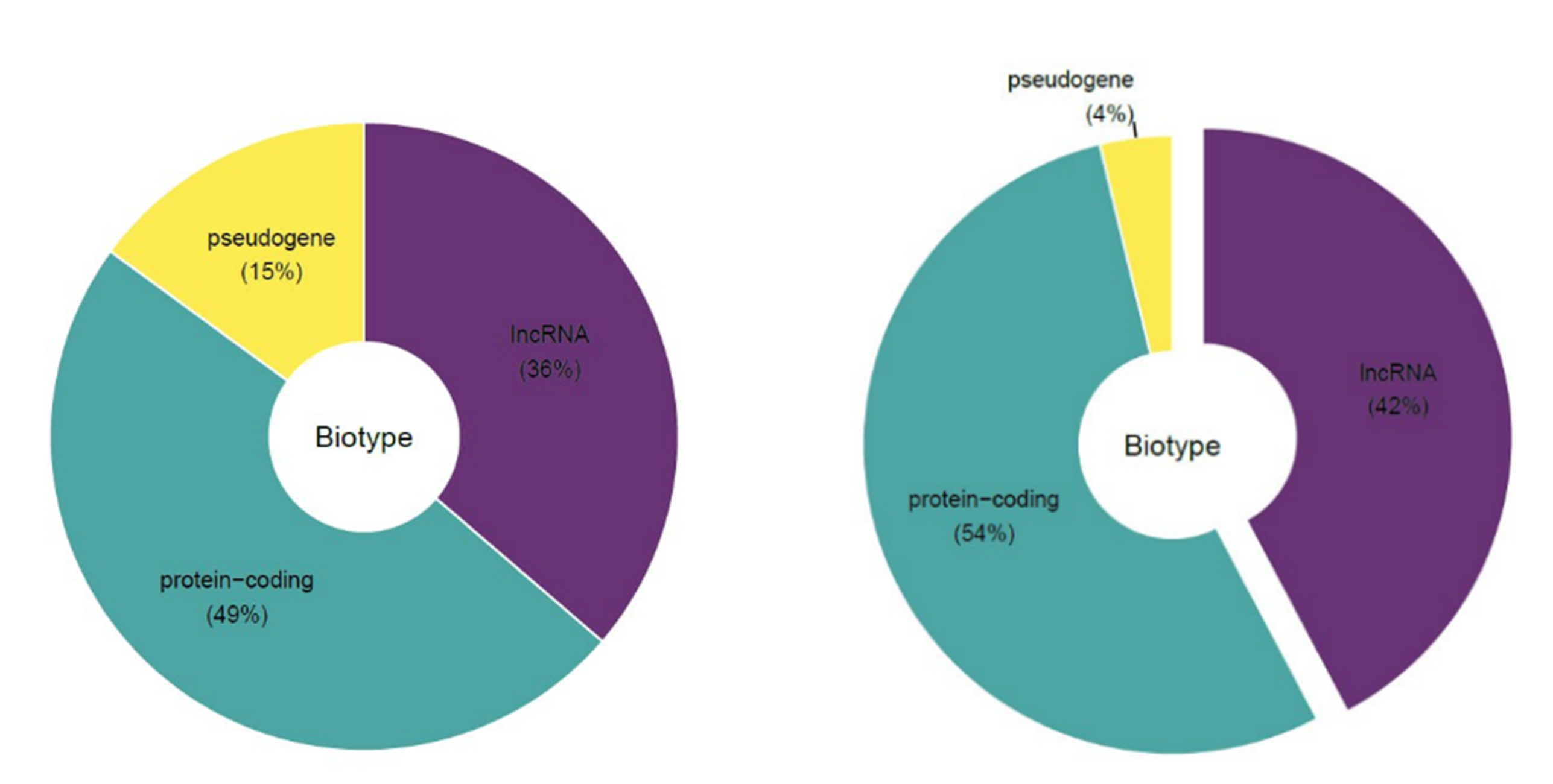

Supplement: S1 Fig — The left pie chart shows RNA biotypes of all expressed transcripts, while the right pie chart displays RNA biotypes of differentially expressed transcripts. (TIF) [file pone.0326901.s001.tif]

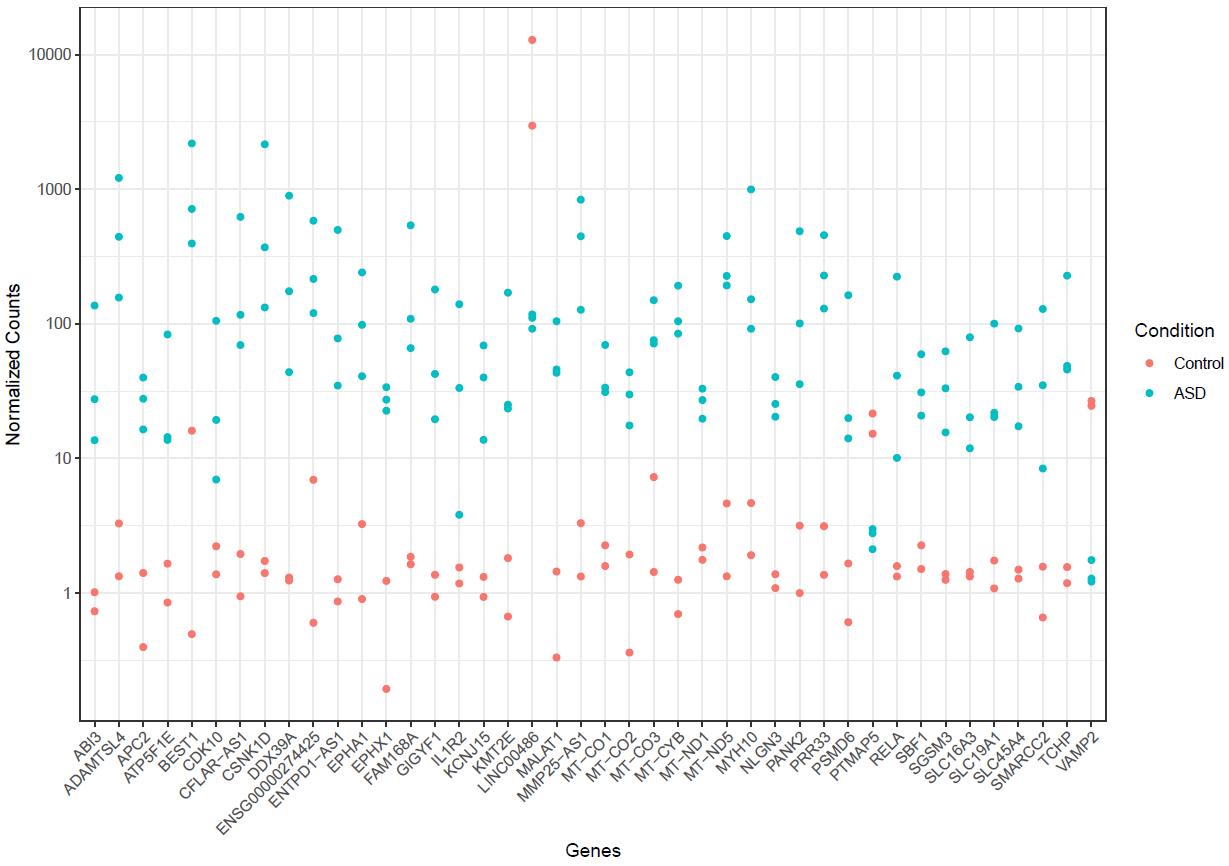

Supplement: S2 Fig — The graph is based on normalized transcript counts. Blue and red bars represent autistic patients and healthy controls, respectively. (TIF) [file pone.0326901.s002.tif]

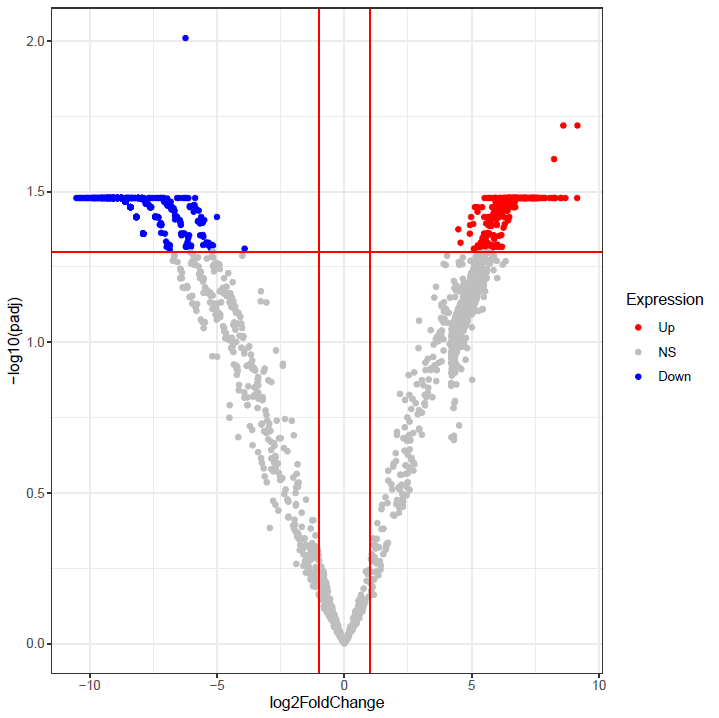

Supplement: S3 Fig — The plot displays the relationship between log₂ fold change and statistical significance (adjusted p-value). The x-axis represents the log₂ fold change in transcript expression, while the y-axis shows the –log₁₀ of the adjusted p-value. Transcripts with significant differential expression (Padj < 0.05) are highlighted in red for those upregulated in autistic patients (log₂ fold change > 2) and in blue for those downregulated (log₂ fold change < –2). (TIF) [file pone.0326901.s003.tif]

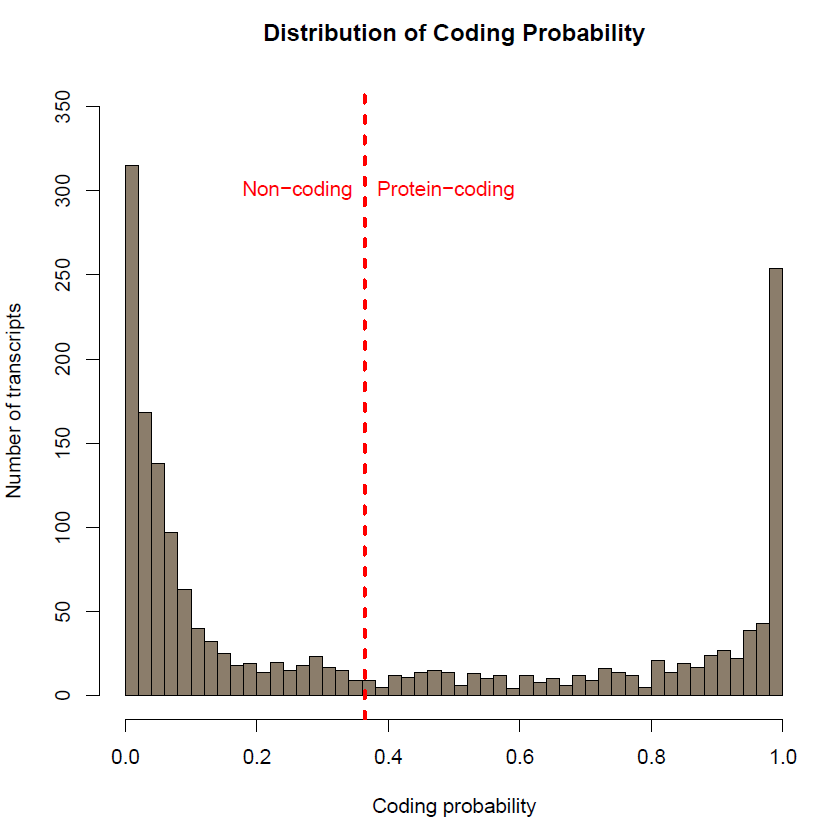

Supplement: S4 Fig — Graph shows number of transcripts per coding probability. The dashed red line shows the threshold (0.364). Coding probability below this amount considered non-coding transcripts. (TIF) [file pone.0326901.s004.tif]

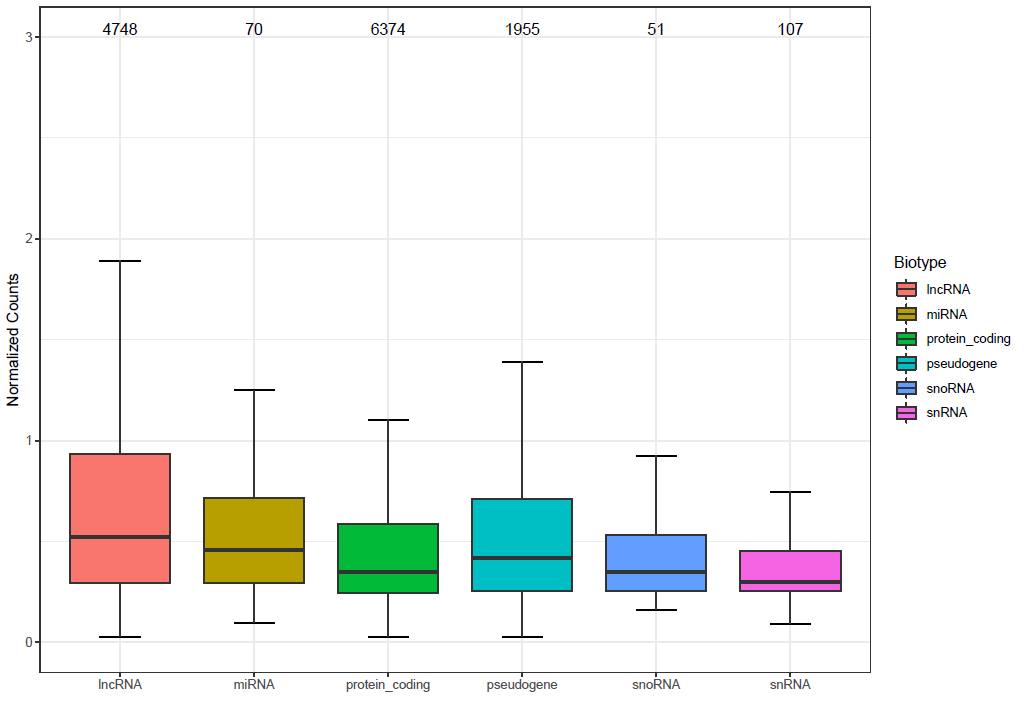

Supplement: S5 Fig — Expression values are log2- normalized counts. The top of each bars shows the number of each transcript per genomic regions. (TIF) [file pone.0326901.s005.tif]

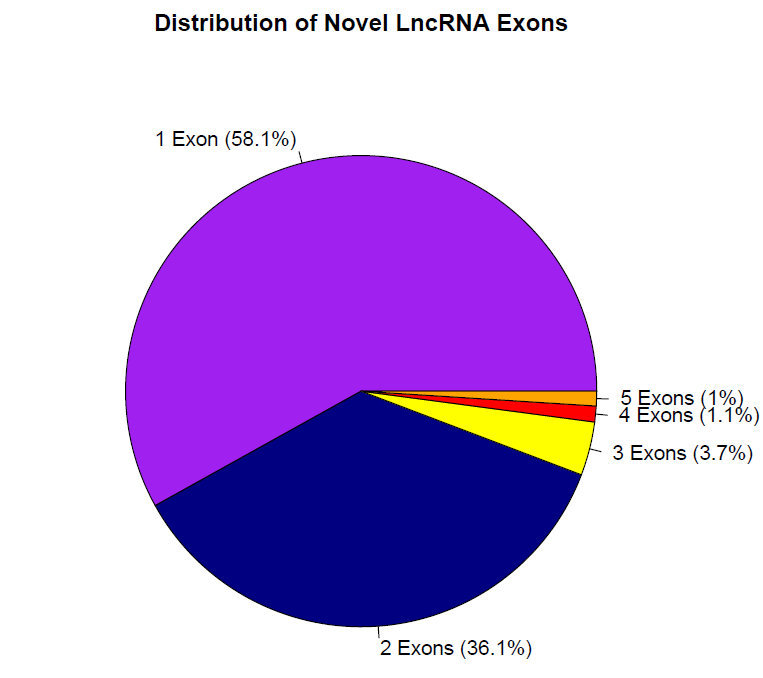

Supplement: S6 Fig — 94.2% of the lncRNA transcripts had either 1 or 2 exons. (TIF) [file pone.0326901.s006.tif]

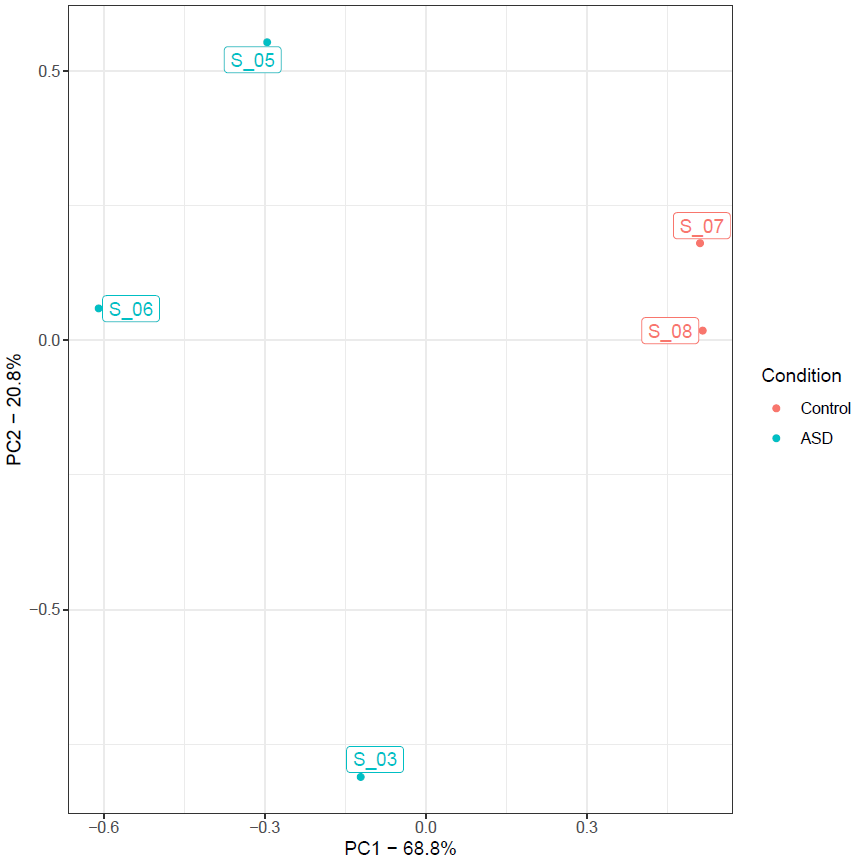

Supplement: S7 Fig — The first principal component (PC1) explains 68.8% of the variance, while the second component (PC2) explains 20.6%. Blue points represent ASD samples (S_03, S_05, S_06), and red points represent control samples (S_07, S_08), indicating distinct clustering between the two conditions. (TIF) [file pone.0326901.s007.tif]

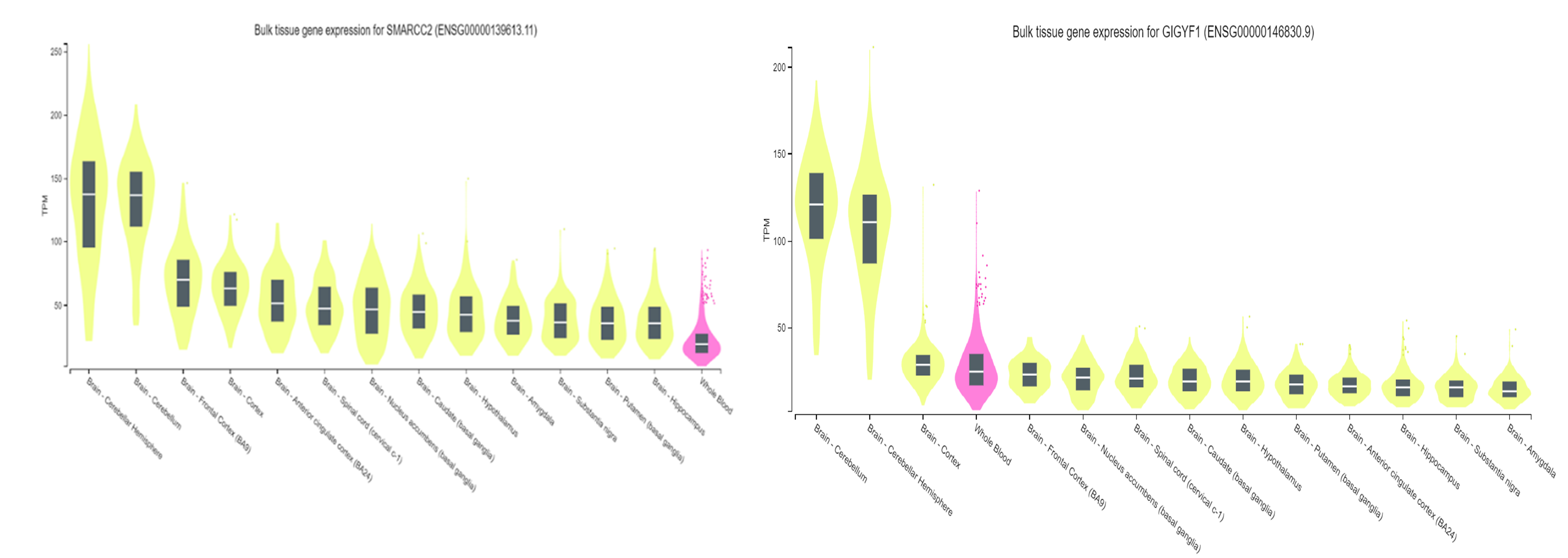

Supplement: S8 Fig — Notably, both genes show significant expression in whole blood. (TIF) [file pone.0326901.s008.tif]

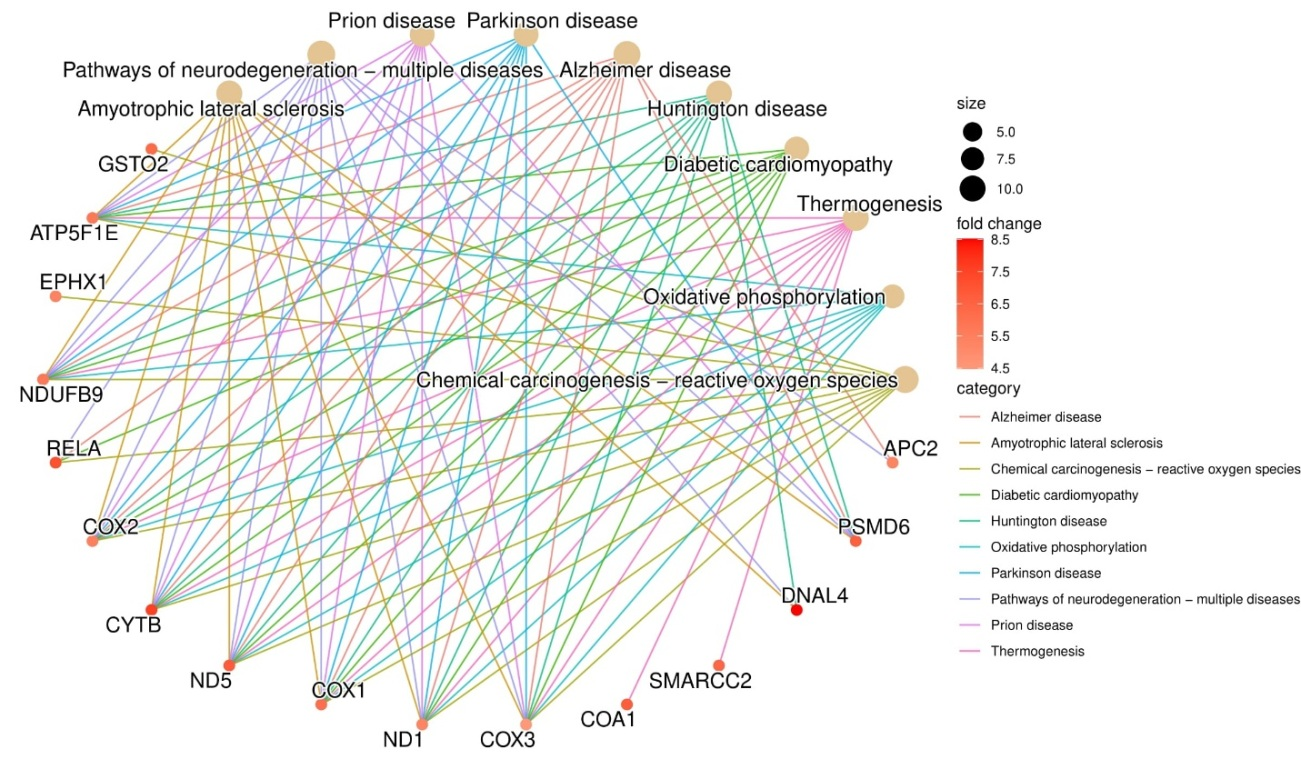

Supplement: S9 Fig — Various genes of different mitochondrial electron transport chain complexes are present. The size of each circle representing GO terms indicates the number of genes associated with that term. Fold change (log2) is based on the normalized counts of each gene. (TIF) [file pone.0326901.s009.tif]
