# Supplementary material for: DNA/RNA hybrid profiling in autistic patients: A focus on mRNA and non-coding RNA variations
Source: PLoS One. 2025 Nov 3;20(11):e0326901. doi: 10.1371/journal.pone.0326901 (PMC12582435; doi:10.1371/journal.pone.0326901)
Supplement: S1 Table — (PDF) [file pone.0326901.s010.pdf]

S1 Table. Clinical characteristics of patients and healthy controls, including age and gender distribution

Samples	Age	Gender
Patient-1 (S1)	3 years	Male
Patient-2 (S2)	9 years	Male
Patient-3 (S3)	5 years	Male
Patient-4 (S4)	3 years	Female
Patient-5 (S5)	12 years	Female
Patient-6 (S6)	6 years	Female
Control-1 (S7)	9 years	Female
Control-2 (S8)	6 years	Female
Control-3 (S9)	8 years	Female
Control-4 (S10)	5 years	Male
Control-5 (S11)	8 years	Male
Control-6 (S12)	14 years	Male
