# Supplementary material for: DNA/RNA hybrid profiling in autistic patients: A focus on mRNA and non-coding RNA variations
Source: PLoS One. 2025 Nov 3;20(11):e0326901. doi: 10.1371/journal.pone.0326901 (PMC12582435; doi:10.1371/journal.pone.0326901)
Supplement: S2 Table — (PDF) [file pone.0326901.s011.pdf]

**S2 Table. Sensitivity and precision percent across multiple levels of transcripts**

| Levels | Sensitivity | Precision |
| --- | --- | --- |
| Base level | 100% | 94.5% |
| Exon level | 93.2% | 95.6% |
| Intron level | 100% | 98.5% |
| Intron chain level | 100% | 95.6% |
| Transcript level | 99.5% | 94.2% |
| Locus level | 99.6% | 92.1% |
