# Supplementary material for: DNA/RNA hybrid profiling in autistic patients: A focus on mRNA and non-coding RNA variations
Source: PLoS One. 2025 Nov 3;20(11):e0326901. doi: 10.1371/journal.pone.0326901 (PMC12582435; doi:10.1371/journal.pone.0326901)
Supplement: S5 Table — The cause of the genetic association of each gene with ASD and the number of reports for each gene in relation to ASD is also present. (PDF) [file pone.0326901.s014.pdf]

S4 Table. List of ASD-related genes among the DEGs, previously reported in the SFARI database as being associated with autism. The cause of the genetic association of each gene with ASD and the number of reports for each gene in relation to ASD is also present

Gene symbol	Genetic category	Number of reports
EPHA1	Rare Single Gene Mutation	6
GIGYF1	Rare Single Gene Mutation	14
IL1R2	Rare Single Gene Mutation	6
KCNJ15	Rare Single Gene Mutation	3
KMT2E	Rare Single Gene Mutation, Syndromic, Genetic Association	16
MYH10	Rare Single Gene Mutation	5
NLGN3	Rare Single Gene Mutation, Genetic Association, Functional	39
SBF1	Rare Single Gene Mutation	9
SGSM3	Rare Single Gene Mutation	6
SMARCC2	Rare Single Gene Mutation, Syndromic, Functional	15
