# Supplementary material for: DNA/RNA hybrid profiling in autistic patients: A focus on mRNA and non-coding RNA variations
Source: PLoS One. 2025 Nov 3;20(11):e0326901. doi: 10.1371/journal.pone.0326901 (PMC12582435; doi:10.1371/journal.pone.0326901)
Supplement: S6 Table — (PDF) [file pone.0326901.s015.pdf]

S3 Table. List of primers sequencing nucleotides forward and reverse for Real-Time PCR

Oligo name	5` - Oligo Seq - 3`
Hsa_SLC12A5-AS1-F	CCTGAATCTGGCCACTTCGC
Hsa_SLC12A5-AS1-R	CTCCTTCAGTACAGGACGGC
Hsa_RN7SK-F	CATCCCCGATAGAGGAGGACC
Hsa_RN7SK-R	ATGCAGCGCCTCATTTGGATG
Hsa_SLC16A3_F	CCACAAGTTCTCCAGTGCCATTG
Hsa_SLC16A3_R	CGCCAGGATGAACACGTACATG
Hsa_NLGN3-F	CGGGTTGGAGTGCTAGGTTT
Hsa_NLGN3-R	ATATTCTCGCTCACCCAGCG
Hsa_SMARCC2-F	CTGTGGCTCGGCAAGAACTA
Hsa_SMARCC2-R	GAAATCGTAACGCCGCCATC
Hsa_ADAMTSL4-F	AACTACCTGGCACTTCGTGG
Hsa_ADAMTSL4-R	ATATCGAAAGACGGTCCCGC
